# Reintroducing Flax (*Linum usitatissimum* L.) to the Mediterranean Basin: The Importance of Nitrogen Fertilization

**DOI:** 10.3390/plants10091758

**Published:** 2021-08-24

**Authors:** Ioanna Kakabouki, Antonios Mavroeidis, Alexandros Tataridas, Ioannis Roussis, Nikolaos Katsenios, Aspasia Efthimiadou, Evangelia L. Tigka, Stella Karydogianni, Charikleia Zisi, Antigolena Folina, Dimitrios Bilalis

**Affiliations:** 1 Laboratory of Agronomy, Department of Crop Science, Agricultural University of Athens, 11855 Athens, Greece; antoniosmauroeidis@gmail.com (A.M.); a.tataridas@gmail.com (A.T.); iroussis01@gmail.com (I.R.); stella.karidogianni@hotmail.com (S.K.); xarikleiazisi@gmail.com (C.Z.); folinanti@gmail.com (A.F.); bilalisdimitrios@gmail.com (D.B.); 2 Institute of Soil and Water Resources, Department of Soil Science of Athens, Hellenic Agricultural Organization DEMETER, Sofokli Venizelou 1, 14123 Lykovrissi, Greece; nkatsenios@gmail.com (N.K.); sissyefthimiadou@gmail.com (A.E.); evitiga@yahoo.gr (E.L.T.)

**Keywords:** Accumulated growing-degree days (AGDDs), flax, nitrogen fertilization, nitrogen indices, Mediterranean basin, climate change

## Abstract

An increasing interest has been reported regarding the reintroduction of flax in the Mediterranean region. The aim of this present study was to evaluate the effects of nitrogen (N) fertilization on the performance of flax cv. Everest, under Mediterranean climate conditions. A two-year study was carried out in 2018–2019, in Western Greece. The experiment was set-up in a randomized complete block design with four replications and six treatments of different N fertilization rates (0, 20, 30, 40, 50, and 60 kg N ha^−^^1^). Measurements included plant biomass, the leaf area index (LAI), the yield, and the Growth Degree Days (GDDs) required for full seed maturity. The N uptake of flax was also evaluated utilizing the Nitrogen Harvesting (NHI) and Nitrogen Utilization Efficiency (NUtE) indices. Although the highest fertilization rate (60N) increased the yield by 35.4% (2018) and 23.1% (2019), a GDDs and N indices assessment revealed that it noted the lowest efficiency and may lead to significant yield losses, as it significantly prolonged the crop cycle. On the contrary, even though fertilization rates of 20 and 30 kg N ha^−1^ increased the yield only by 7% and 15% (on average), they were more efficient, and prolonged the crop cycle less (compared to 60N).

## 1. Introduction

Flax (*Linum usitatissimum* L.), one of the 150 species of the Linaceae family, is a crop with great potential due to its multiple uses and its high adaptability [1,2]. Although it originates from around the Mediterranean region and Southwestern Asia [3], this adaptability to various climatic conditions spread its cultivation across the Middle East, India, Canada, and several European countries [2]. Amongst them, Canada is considered the main producer of flax [4]. Flax is cultivated either for its fiber, or its seeds and the oil produced from them [2]. In fact, flax varieties can be distinguished by either oilseed or fiber varieties [5]. The oil produced from oilseed varieties was initially used as raw material for varnish, paint, ink, and soap production [6], but, nowadays, it is included in human nutrition [7]. According to Bilalis et al. [8], flax oil is placed amongst nature’s best sources of omega-3- fatty acids, while Madhusudhan [4] considered it as nature’s richest source of a-linolenic acid. Furthermore, flaxseed is rich in proteins and dietary fibers, while it has been estimated that 100 g of flaxseed contain 450 kcal [8]. On the contrary, fiber varieties are cultivated for industrial purposes [9,10]. Having been used ever since Neolithic times, flax fibers have reported a broad spectrum of applications, from human clothing to the creation of synthetic materials [1,11]. Flax can also be included in animal rations [12] or be used for the phytoremediation of metal-contaminated soils [10].

Throughout recent decades, an increasing interest regarding the reintroduction of flax has been reported in the Mediterranean region [13]. This increasing interest could partially be attributed to the adverse effects of climate change as the introduction of alternative crops, such as flax, to the Mediterranean countries could potentially tackle this rising problem [14]. Studies have been conducted in Italy and Spain in order to investigate the influence of sowing and harvest time, as well as irrigation, on fiber flax varieties [13,15]. However, fertilization could be the determinant factor for the successful reintroduction of flax in the Mediterranean Basin as the literature indicates that, besides tillage [8,16], the factor that affects yield, seed, and fiber quality most is fertilization [6,17,18], especially nitrogen (N) fertilization [19]. N is known to be one of the most important nutrients for plant growth. Flax, among many other crops, is positively affected by increased N supply. Rahimi et al. [20] reported that the application of Ν fertilization on flax results in high yields and high-quality flaxseed. However, an N oversupply can delay crop maturation, as it promotes vegetative growth and potentially reduces yield. According to Franzen [21], this negative impact of excessive N fertilization has also been reported on flax cultivations. Many studies have aimed to define the optimum amount of applied N fertilization on flax, yet the results remain contradictory [22].

Nitrogen indices can facilitate the estimation of the optimal dose of nitrogen for each crop. One of the most important N indices is the Nitrogen Harvest Index (NHI), an index that describes the distribution of nitrogen in plant tissues [23]. In addition, Nitrogen Utilization Efficiency (NUtE) is another index that describes the amount of the ground-absorbed N that has been utilized by the plant for seed production [24]. These indices allow us to estimate the nitrogen losses, as well as the amount of nitrogen that has been absorbed by the crop [25] and, thus, the efficiency of the fertilization rate. However, these indices do not assess the overall performance of the crop.

A very useful index for the prediction of the crop performance is Growth Degree Days (GDDs) [26]. GDDs represent the sum of degrees over a temperature threshold in a defined period of time [27]. According to studies performed on crops such as wheat and corn, GDDs represent the amount of heat required by the crops in order to develop [28,29]. Therefore, GDDs could be perceived as a means to describe the duration of the biological cycle of the crop. Regarding the GDD requirements of flax, they have been estimated at 1150 °C for full seed maturity [30]. This roughly translates to 100–120 days after sowing (DAS) [30], or approximately five to six weeks after flowering [31]. It should be noted though that high temperatures and water supply, as well as the density of plants, can delay the maturation of crops. Therefore, environmental factors [32,33,34] and cultivation techniques [35] can differentiate the biological cycle length of this crop.

To our knowledge, the number of studies that have evaluated the effects of N fertilization on the performance of flax, utilizing GDDs and N indices, are limited. Most studies in the literature refer to the effects of nitrogen fertilization on the characteristics of the flaxseed. Thus, this study set two aims. The first one was to examine the effects of different N fertilization levels on the performance of this crop (yield and biological cycle), under Mediterranean conditions. The second one was to evaluate the efficiency of those fertilization levels. In order to perform these tasks, three indices were utilized: LAI, AGDDs, and NHI and NUtE.

## 2. Results

### 2.1. Plant Biomass (DM)

**Figure 1:**
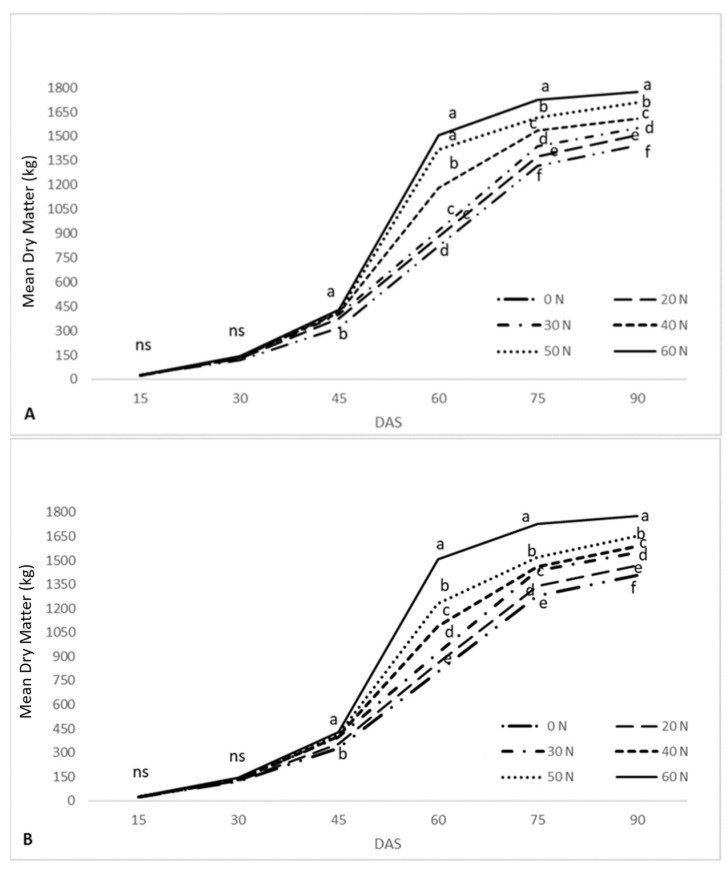
Changes in DM at different DAS (“ns”: not statistically; different letters are significantly different at *p* = 0.05), during 2018 (**A**) and 2019 (**B**).

The fertilization-induced changes in the mean dry weight of flax are presented in Figure 1. In particular, Figure 1A depicts the biomass changes that were recorded during the first year (2018), whereas Figure 1B presents the results of the second year (2019). In 2018, the differences noted amongst the treatments during the first 30 DAS were insignificant. On the contrary, 45 DAS, significant differences were observed between the control (0N) and the treatments (20N, 30N, 40N, 50N, and 60N), although the differences amongst the treatments were trivial. Notably, DM was increased by 35% in 60N, compared to the control. Although DM was significantly increased in all of the treatments 60 DAS, the differences between 50N and 60N were insignificant. Following the 75th DAS, all treatments reported statistically significant differences amongst them. Throughout 2018, the lowest DM values were reported in the control and the highest in 60N. In particular, plant biomass in 60N was increased by 21.22%, 82.07%, and 23.05% 30, 60, and 90 DAS, respectively, compared to the control (Figure 1A).

The results of the second year (Figure 1B) were in agreement with those of 2018. Notably, the average DM in all treatments was reduced, compared to the results of 2018. Following the 45th DAS, plant biomass reported statistically significant differences amongst the treatments. Similar to the findings of the previous year, plants in the control reported the lowest DM values throughout the duration of the experiment. The greatest DM increment (compared to the control) was once again noted in 60N (18.1%, 67.44%, and 21.47% 30, 60, and 90 DAS, respectively). Overall, the biomass of flax was positively correlated to N fertilization as higher fertilization rates resulted in significantly higher biomass accumulation.

### 2.2. Leaf Area Index (LAI) and Accumulated Growth Degree Days (AGDDs) 

LAI values ranged from 1.857 to 2.372, during 2018, and from 2.007 to 2.372, during 2019 (Table 1). Although LAI values were positively correlated to N fertilization, no statistically significant differences were noted between the control and 20N, the 30N and 40N, and the 50N and 60N. The maximum LAI values were recorded in the 60N during both years (2.372) and were by 27.7% (during 2018) and 18.2% (during 2019) higher than the respective control LAI values. The AGDDs were significantly affected by N fertilization (Table 1). In particular, the application of 20 additional kg N ha^−1^ seemed to significantly increase the AGDD requirements of flax in a season. On the contrary, the application of 10 additional kg N ha^−1^ did not affect the AGDDs, as the differences between the 20N and 30N, the 30N and 40N, the 40N and 50N, and the 50N and 60N were not significant. The 60N treatment resulted in the most notable increase in AGDDs, as they were higher by 5.3% and 8.4% (during 2018 and 2019, respectively) compared to 0N.

### 2.3. Plant Height and Yield

Fertilization considerably affected plant height, as statistically significant differences were noted amongst all treatments, during both years. Besides the control, the plant height in the rest of the treatments was higher during 2018. Even though the plant height was significantly increased in all of the treatments (Table 1), the most notable height increment was observed in 60N (43.3% and 31.9% compared to the 0N, during 2018 and 2019, respectively). The results, regarding plant height, indicate that for every 10 additional kg N ha^−1^, plant height increases by 4–9%. Flaxseed yield was also positively affected by N fertilization, although the differences among the higher fertilization rates (between 60N and 50N, and between 50N and 40N) were insignificant. In particular, the yield in 60N was only 2–4% higher than the respective one of 50N. The highest yield was recorded in 60N, where it was increased by 35.4% and 23.1% (compared to 0N), during 2018 and 2019, respectively (Table 1).

### 2.4. 1000 Seed Weight and N Content of the Seeds

The average weight of 1000 seeds was significantly higher during the second experimental year (Table 1). Besides 20N, the average weight of 1000 seeds was 1.7–2.8% higher during 2019. As with the yield response to N fertilization, the weight of 1000 seeds was positively affected by fertilization. It should be noted though that the differences between 40N and 50N, and between 50N and 60N were insignificant. The best results were once again observed in 60N where the 1000 seed weight was 12.3% and 11.6% (during 2018 and 2019, respectively) higher, compared to 0N. In contrast to the 1000 seed weight results, the seed N content did not note significant differences between 2018 and 2019 (Table 1). The highest seed N content was reported in 60N (12.9% and 9.7% higher than the respective one in 0N, during 2018 and 2019, respectively), although the differences between 50N and 60N were trivial.

### 2.5. Above-Ground, Seed, and Total N Uptake 

The Above-ground N Uptake of the plants was significantly increased through each treatment (Table 2). In particular, the 20N, 30N, 40N, 50N, and 60N increased, on average, the respective Above-ground N Uptake of the crop by 10.5%, 20.5%, 33.3%, 45.1%, and 56.7%, respectively, compared to the control (0N). Similarly, the Seed N Uptake increased with fertilization rates, although the differences amongst the higher fertilization rates (40N and 50N, and 50N and 60N) were insignificant (Table 1). During 2018, 40N, 50N, and 60N increased Seed N Uptake (compared to 0N) by 40.2%, 47.3%, and 52.8%, respectively, while during 2019, the same treatments increased the Seed N Uptake by 21.9%, 28.6%, and 35.1%. The Total N Uptake was also positively affected by fertilization. This positive correlation between Total N Uptake and fertilization was anticipated due to the fertilization-induced increment in the Above-ground and Seed Uptake. The highest Total N Uptake was recorded in 60N during both years (53.6% and 37.9% higher compared to 0N, during 2018 and 2019, respectively), followed by 50N (47.2% and 30.7%), 40N (39.1% and 23.6%), 30N (24.7% and 16%), and 20N (11.1% and 8.5%) (Table 2).

### 2.6. Nitrogen Harvest Index (NHI) and Nitrogen Utilization Efficiency (NUtE) 

The values of the nitrogen harvest index (NHI) ranged from 0.845 to 0.857 during the first year, and from 0.845 to 0.863 during the second year. The highest NHI value was recorded in the 40N during 2018 (0.857), and in the control (0N) during 2019 (0.863). The lowest NHI values were recorded in 60N during both years (0.845). It should be noted though that the differences between the treatments were found statistically insignificant during both years (Table 2). On the contrary, the NUtE values significantly decreased with the fertilization rates. Therefore, the highest NUtE values were reported in the control during both years (Table 2) and the lowest in the 60N (reduced by 11.8% and 10.7% compared to 0N, during 2018 and 2019, respectively). The lower fertilization rates (20N and 30N) did not report any statistically significant differences amongst them.

## 3. Discussion

The results of our study initially indicated a positive correlation between AGDDs, the agronomic characteristics of flax, and the weight of 1000 seeds (Table 3). Similar findings have been reported by Mirshekari et al. [36], though in their research, the positive correlation between GDDs and seed traits was attributed to the difference in sowing days. According to Mirshekari et al. [36], early or late sowing dates affect day length, photoperiod, and other factors that may or may not cause abiotic stress on the plants and, therefore, alter the growing cycle of flax and potentially increase or decrease yield. In this present study, AGDDs were utilized as a mere indicator of the effects of N fertilization on the length of the growing cycle of the crop. Indeed, our results reveal that under the same edaphoclimatic conditions, the application of N fertilization increases the AGDDs (by 74 and 113 GDDs on 2018 and 2019, respectively) required for full seed maturity; thus, it is safe to assume that providing additional N to flax prolongs the vegetive phase of the plant and delays seed maturity, as Franzen [21] proposed. Therefore, the positive correlation between AGDDs, and plant height and biomass (Table 3) can be interpretated as the result of a longer vegetative phase that led to larger stems, more branches, and enhanced canopy. 

Regarding the effects of N fertilization on the performance of flax, as presented in Table 1 and Figure 1, the application of N fertilization clearly affected the agronomic characteristics (height and the biomass) of the plants. This positive response of height to N fertilization has been reported before. In particular, Ali et al. [37] observed that the application of 75 kg ha^−1^ of N fertilization increased the height of the plants by approximately 15.6%. In contrast to the findings of Ali et al. [37], in our study, the height of plants treated with 30 kg N ha^−1^ (30N) was increased by 16.45% (average height increase of both years), indicating that lower levels of N fertilization led to greater increases in the height of the plants. This dissimilarity could be attributed to different initial levels of soil N. This hypothesis could also explain the contradictory findings of Kariuki et al. [38], as their results found no significant interaction between fertilization and plant height. Other potential explanations include N leaching and genetic factors, as the varieties of flax used on each occasion were different. 

The augmentation of the biomass (DM) of the plants due to the application of N fertilization has also been reported by other researchers. Soethe et al. [39] noted a positive linear increase while examining the interaction of N fertilization and the biomass of flax plants. In our study, during both experimental years, the dry weight of the plants was significantly affected by fertilization, particularly on treatments with higher fertilization rates (Figure 1). This positive interaction between fertilization and biomass could initially be attributed to the aforementioned prolongation of the vegetive phase that results in the formation of more above-ground plant organs (e.g., leaves and branches) compared to untreated plants. Furthermore, studies have suggested that N affects the photosynthetic processes of the plants [40] due to its importance for the Rubisco carboxylating enzyme [41,42,43] and chlorophyll a and b [44,45]. In fact, according to Yu et al. [46] and Maleva et al. [47], higher contents of chlorophyll a and chlorophyll b ensure higher plant biomass. The increased LAI values recorded in the present study (Table 1) also indicate increased fertilizer-induced photosynthetic activity. Finally, it should be noted that, as presented in Figure 1, a significant increment in flax dry weight was observed following the 45^th^ DAS when the canopy of the plants was well-developed [30] and photosynthetic activity was arguably more intense. 

The yield was also significantly increased as fertilization levels were increased. Studies in the literature also support our results as N fertilizations reportedly increase flaxseed yields [21,38,48]. According to Kariuki et al. [38], N fertilization increases vegetative growth, which results in a greater number of capsules per plant and, therefore, each plant produces more seeds. In our study, the application of 130 kg of urea per ha (60N) resulted in the greatest increment in seed production as it increased flaxseed yield by 35.4% and 23.1% in 2018 and 2019, respectively. Notably, these results contradict the findings of Franzen [21] as he stated that fertilization rates greater than 56 kg N ha^−1^ (for no-till production) tend to reduce yields on flax due to the greater susceptibility to diseases and lodging. Such phenomena were not observed at significant rates throughout our study. In fact, a wide range of fertilization rates (20–120 kg N per ha) have been proposed on flax cultivations throughout the world, and in certain instances, they far exceed Franzens’ proposition [19,49,50,51]. These contradictions are sensible as soil properties and soil fertility vastly differentiate on each occasion and, thus, these factors should always be considered [21]. Consequently, the amount of additional N provided through fertilization should perhaps be evaluated alongside the efficiency of the fertilization.

In order to perform this evaluation, two nitrogen indices were utilized in our study: NHI and NUtE. For the estimation of NUtE, the Above-ground N uptake (ANU), Seed N uptake (SNU), and Total N uptake (TNU) had to be measured first. ANU, SNU, and TNU were found positively correlated with N fertilization as their values increased with fertilization rates (Table 2). The observations regarding ANU and TNU were anticipated as the biomass and the N content of the plants were affected by the fertilization, as mentioned above. The increased values of SNU, however, and especially the increment in N% of the seeds, are in disagreement with the literature. According to Rahimi et al. [20], as the yield increases, the N content of the seeds tends to decrease, as these factors are negatively correlated. Moreover, N fertilization is believed to impede the seed-filling of various crops [52]. However, studies have indicated that the N content of flaxseed is negatively correlated with their oil content [53,54]. This equilibrium between N and oil content could perhaps justify our results as studies in the literature have suggested that high N fertilization rates negatively affect the oil content of the seed [22,55,56]. This phenomenon is called the “dilution effect” and is the result of the oil-diluting, protein and starch accumulation in the seed due to the N-induced promotion of the vegetative growth that delays grain filling and grain maturity [57]. It should be noted though that the oil content of the seeds was not measured in the present study.

The values of NUtE, in contrast to those of ANU, SNU, and TNU, were negatively correlated with N fertilization rates (Table 2). Furthermore, NHI was not significantly affected by fertilization (Table 2). Both of these indices were also found negatively correlated with the agronomic characteristics of the plants and the weight of 1000 seeds (Table 3). Based on these observations, it is safe to assume that although N fertilization improved the overall performance of flax, higher rates of fertilization were less efficient, probably due to N losses. These losses were potentially caused by nitrate leaching [58] or ammonia volatilization [59]. N losses are a well-known problem of modern-age agriculture with severe environmental imprints [60,61,62]. The economic and ecological aspects of fertilization need to be addressed as our results suggest a noteworthy inefficiency of urease fertilization. Although the application of 130 kg ha^−1^ (60N) of urease fertilizer resulted in a sufficient increment in yield, perhaps the use of slow-release fertilizers should be considered on flax crops. Experiments conducted by Kakabouki et al. [63] regarding the application of slow-release fertilizers with urease and nitrification inhibitors on flax were encouraging. Nevertheless, further research should be conducted regarding this aspect.

Overall, despite the positive response in yield, higher urea fertilization rates seem inadequate. Besides the aforementioned reduced efficacy of these rates, the prolongation of the biological cycle of the crop might also result in reduced yields and inadequate seed quality in the near future. As the adverse effects of climate change are evident in the Mediterranean Basin, the mean temperature is expected to rise even more in the future [64,65]. Despite the great acclimatization potential of flax, temperatures over 30 °C and precipitations over 700 mm per year do not favor its growth [12]. Figure 1 depicts that during the summer months, mean temperatures rise close to 30 °C. According to Kraft et al. [66], exposing flax to temperatures above 30 °C for more than 5 days during the period from flowering to complete seed maturity results in reduced seed production. Similarly, Gusta et al. [67] stated that in the absence of drought stress, exposure to temperatures over 30 °C for 7 days reduces seed production by 30%. Therefore, as high N fertilization levels delay harvest, they could potentially lead to yield losses in the near future due to the high temperatures of the summer months in the Mediterranean Basin. Early sowing could be a possible solution to this problem; nevertheless, further research should be conducted on the effects of sowing dates and fertilization rates on the yield of flax under the Mediterranean climate conditions.

## 4. Materials and Methods

### 4.1. Experimental Design

**Figure 2:**
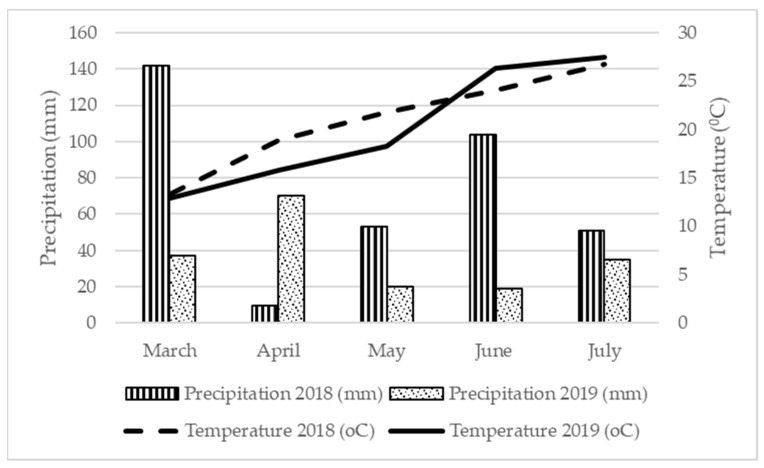
Mean monthly temperature (°C) and precipitation (mm) recorded on the experimental fields throughout the duration of the experiments (March–July, 2018–2019).

The experiment took place in 2018, in Agrinio, Western Greece (38°33′ N, 21°24′ E), and was then repeated for a second year (2019) in the same field. The soil properties of the field are presented in Table 4. The measurements were conducted de novo and in the Laboratory of Agronomy at the Agricultural University of Athens (37°59′01.1′′ N 23°42′12.0′′ E). The forecrop was maize. Each year, three weeks prior to the sowing, the stale seed bed technique was applied on the field. During this period, once a week, a light irrigation was performed in order to stimulate weed emergence. Weeds were then removed by hand. Similarly, throughout the duration of the experiment, the field was weekly inspected, and weeds were removed by hand. Hand weeding was favored over chemical management as several herbicides (pendimethalin, MCPA, Dicamba) that are widely used in Greece (both pre- and post-emergent) have been reported to injure flax [68,69,70]. For the establishment of the experimental field, 300 seeds of the oil variety Everest (*Linum usitatissimum* L. cv. Everest) per m^2^ were sown by hand at a depth of 1 cm and with a 20 cm row spacing. On the first year, the sowing was performed on 22 March (2018), and on the second, on 26 March (2019). The experimental area was, in total, 480 m^2^. The experiment was set-up in a Complete Randomized Block Design (CRBD) with four replications and six different treatments (0N/Control, 20N, 30N, 40N, 50N, and 60N); therefore, each plot was 20 m^2^. On each treatment, a different rate of fertilization was applied (Table 5). The fertilizer used was NUTRIPLUS (urea based, N-P-K: 46-0-0), by Phytothreptiki S.A. (Ano Liosia, West Attica, Greece). According to the literature, when flax is cultivated under a no-tillage system, fertilizers should be broadcasted prior to the sowing [71]; thus, each year, 3 days prior to the sowing, the fertilizer was broadcasted, and the field was irrigated. Following the crop establishment, the field was irrigated twice each year (once during flowering and once during early pod filling) through a sprinkler irrigation system that provided a total of 250 mm of water per irrigation. The meteorological data (mean temperature, precipitations) recorded during these two years are presented below (Figure 2) and were derived from the weather station in Agrinio of the National Observatory of Athens. The experiment ended on June 30 in 2018, and on 4 July in 2019.

### 4.2. Measurements

The biomass of the plants (DM) was measured at 15, 30, 45, 60, 75, and 90 DAS and the height of the plants was measured 90 DAS. For the estimation of the plant biomass, three 0.25 m^2^ quadrats were placed randomly in each plot. The plants within these plots were clipped at ground level and placed in an oven at 80 °C for 72 h. The Leaf Area Index (LAI) was measured using an automatic leaf area meter (Delta-T Devices Ltd., Cambridge, UK) 60 DAS, at the fast vegetation-growth stage [30]. The yield (kg ha^−1^) and the weight of 1000 seeds (g) were measured on the day of the harvest. Each plot was harvested at full seed maturity (seed moisture was at 13%) and the harvest was conducted by hand.

The N content of the above-ground plant tissues and the seeds was measured by applying the Kjeldahl method [72], using a Buchi 316 device (BÜCHI Labortechnik AG, Flawil, Switzerland). Following the harvest, 5 dried plant samples and 10 g of seed were collected from each plot, and then grounded to a fine powder in order to estimate their N content. The N uptake of the above-ground plant tissues (Above-ground N uptake) and the seeds (Seed N Uptake) was estimated according to Equations (1)–(3) (Table 6). For the assessment of the N content and N uptake, two nitrogen indices were utilized, the Nitrogen Harvest Index and the Nitrogen Utilization Efficiency (Table 6). The Nitrogen Harvest Index (NHI) is defined as a ratio of the seed N uptake to the total N uptake of the plant (4) [23]. Nitrogen Utilization Efficiency (NUtE) is defined as the ratio of the yield to the total N uptake (5) [24]. Finally, the Accumulated Growth Degree Days (GDDs) were also calculated, from sowing to harvest day, according to Equation (6) (Table 6).

### 4.3. Statistical Analysis 

Analysis of variance was carried out using the Statistica (Stat Soft, 2011) logistic package. Significant differences between treatments were compared using Tukey’s HSD (honestly significant difference) test at the 5% level of probability (*p* < 0.05).

## 5. Conclusions

Flax responds positively to urea-based N fertilization. Our results suggest that high N fertilization rates can potentially increase flaxseed yield even by 35%. These fertilization rates, nevertheless, were found to be inefficient as a significant portion of the fertilizer was not assimilated by the crop (it was probably volatized or leached). Concurrently, the high fertilization rates prolong the crop cycle. This should be taken into account in order to successfully reintroduce flax in the Mediterranean Basin, as flaxseed is susceptible to the high temperatures of the summer months. Even though further research should be conducted, the results of this present study indicate that low fertilization rates (e.g., 20–30 kg N ha^−1^) should be considered when cultivating flax in the Mediterranean region, as the adverse effects of climate change may pose a threat, in the near future, to flaxseed production. 

## Figures and Tables

**Table 1:** The agronomic characteristics of flax, yield, seed N content and uptake, and the AGDDS, as affected by fertilization.

Treatments	LAI	Plant Height (cm)	Yield (kg ha^−1^)	1000 Seed Weight (g)	Seed N Content (%)	Seed N Uptake (kg ha^−1^)	AGDDs
				2018			
0N	1.86 ^a^	50.75 ^a^	1090 ^a^	3.61 ^a^	2.96 ^a^	32.22 ^a^	1406.4 ^a^
20N	1.97 ^ab^	56.00 ^b^	1180 ^b^	3.75 ^b^	3.04 ^b^	35.85 ^b^	1435.3 ^b^
30N	2.10 ^c^	60.25 ^c^	1297 ^c^	3.79 ^c^	3.12 ^b^	40.48 ^c^	1451.8 ^bc^
40N	2.15 ^bc^	63.50 ^d^	1405 ^d^	3.90 ^d^	3.22 ^c^	45.17 ^d^	1464.7 ^cd^
50N	2.27 ^d^	66.75 ^e^	1450 ^de^	3.96 ^de^	3.27 ^cd^	47.45 ^de^	1473.4 ^de^
60N	2.37 ^d^	72.75 ^f^	1476 ^e^	4.05 ^e^	3.34 ^d^	49.24 ^e^	1480.4 ^e^
				2019			
0N	2.01 ^a^	52.50 ^a^	1175 ^a^	3.68 ^a^	2.99 ^a^	35.18 ^a^	1349.5 ^a^
20N	2.04 ^ab^	55.50 ^b^	1231 ^b^	3.75 ^b^	3.09 ^b^	38.05 ^b^	1378.1 ^b^
30N	2.15 ^c^	60.00 ^c^	1298 ^c^	3.89 ^c^	3.12 ^b^	40.52 ^bc^	1402.1 ^bc^
40N	2.24 ^bc^	63.00 ^d^	1348 ^cd^	3.97 ^d^	3.18 ^c^	42.89 ^c^	1425.6 ^cd^
50N	2.3 ^d^	66.50 ^e^	1395 ^de^	4.05 ^de^	3.24 ^cd^	45.24 ^cd^	1437.5 ^de^
60N	2.37 ^d^	69.25 ^f^	1447 ^e^	4.10 ^e^	3.28 ^d^	47.52 ^d^	1462.9 ^e^
F_*Fert*_	56.27 ^***^	169.26 ^***^	132.3 ^***^	57.12 ^***^	70.93 ^***^	162.58 ^***^	40.57 ^***^
F_*Year*_	12.357 ^***^	ns	ns	13.79 ^***^	ns	ns	ns
F_*Fert × Year*_	ns	ns	6.8 ^***^	ns	ns	6.75 ^***^	ns

Means within a column followed by the different letters are significantly different at *p* = 0.05. (“ns”: not statistically significant; ***: statistically significant for a significance level of *p* < 0.001).

**Table 2:** The N content of the above-ground plant tissues, the Above-ground and Total N Uptake, and the N indices, as affected by the fertilization.

Treatments	N Content of the Above-Ground Tissues (%)	Above-Ground N Uptake (kg ha^−1^)	Total N Uptake (kg ha^−1^)	NHI	NUtE
			2018		
0 N	0.40 ^a^	5.70 ^a^	37.92 ^a^	0.849 ^ns^	28.752 ^a^
20N	0.42 ^b^	6.29 ^b^	42.13 ^b^	0.851 ^ns^	28.015 ^b^
30N	0.44 ^b^	6.82 ^c^	47.30 ^c^	0.856 ^ns^	27.434 ^b^
40N	0.47 ^c^	7.57 ^d^	52.73 ^d^	0.857 ^ns^	26.643 ^c^
50N	0.49 ^cd^	8.37 ^e^	55.82 ^e^	0.850 ^ns^	25.979 ^d^
60N	0.51 ^d^	9.01 ^f^	58.25 ^e^	0.845 ^ns^	25.352 ^e^
			2019		
0 N	0.40 ^a^	5.67 ^a^	40.85 ^a^	0.863 ^ns^	28.845 ^a^
20N	0.43 ^b^	6.28 ^b^	44.33 ^b^	0.861 ^ns^	27.869 ^b^
30N	0.45 ^b^	6.88 ^c^	47.39 ^c^	0.856 ^ns^	27.422 ^b^
40N	0.48 ^c^	7.59 ^d^	50.48 ^d^	0.852 ^ns^	26.780 ^c^
50N	0.49 ^cd^	8.14 ^e^	53.38 ^e^	0.848 ^ns^	26.156 ^d^
60N	0.52 ^d^	8.81 ^f^	56.33 ^f^	0.845 ^ns^	25.756 ^e^
F_*Fert*_	38.496 ^***^	96.627 ^***^	218.425 ^***^	2.62 ^*^	125.632 ^***^
F_*Year*_	ns	ns	ns	ns	ns
F_*Fert × Year*_	ns	ns	6.361 ^***^	ns	ns

Means within a column followed by the different letters are significantly different at *p* = 0.05. (“ns”: not statistically significant; *: statistically significant for a significance level of *p* < 0.05; ***: statistically significant for a significance level of *p* < 0.001).

**Table 3:** Correlation matrix among AGDDs, seed traits, yield, agronomic features of flax, and N indicies.

	AGDDs	N% Seed	N% Upper	N Seed	N Upper	N Total	NHI	NUtE
AGDDs	-	0.668 ^***^	0.656 ^***^	0.702 ^***^	0.723 ^***^	0.714 ^***^	−0.410 ^**^	−0.701 ^***^
Plant Height	0.745 ^***^	0.946 ^***^	0.914 ^***^	0.962 ^***^	0.961 ^***^	0.971 ^***^	−0.427 ^**^	−0.964 ^***^
DM	0.769 ^***^	0.904 ^***^	0.873 ^***^	0.918 ^***^	0.961 ^***^	0.934 ^***^	−0.535 ^**^	−0.946 ^***^
Yield	0.695 ^***^	0.942 ^***^	0.895 ^***^	0.994 ^***^	0.924 ^***^	0.992 ^***^	−0.270 ^ns^	−0.929 ^***^
1000 seed weight	0.622 ^**^	0.807 ^***^	0.895 ^***^	0.860 ^***^	0.903 ^***^	0.876 ^***^	−0.513 ^**^	−0.856 ^***^

Significance levels: ** *p* < 0.01; *** *p* < 0.001; ns, not significant (*p* > 0.05).

**Table 4:** Soil properties of the experimental field.

Soil Type	Clay Loam
Clay	29.8%
Silt	34.3%
Sand	35.9%
pH	7.22
Organic matter	2.25%
CaCO_3_	14%
Total Mineral Nitrogen	0.149%
Phosphorus—P Olsen	170 mg kg^−1^ soil
Potassium	625 mg kg^−1^ soil

**Table 5:** Treatments and amount of applied N fertilization.

Treatment	Applied Fertilizer (kg ha^−1^)	Fertilization Rate (kg N ha^−^^1^)
0N (Control)	-	-
20N	43.5	20
30N	65	30
40N	87	40
50N	109	50
60N	130.5	60

**Table 6:** The equations used in the present study and their corresponding number.

Equation	Equation Number	Reference
$Above-ground\;N\;uptake=\frac{DM\;\left(kg\;ha^{-1}\right)*Above-ground\;tissues\;N\;content\;\left(\%\right)}{100}$	(1)	[25]
*Seed N uptake =* $\frac{Seed\;yield*seed\;N\;content\;\left(\%\right)}{100}$	(2)	[25]
*Total N uptake = Above-ground N uptake + Seed N uptake* (kg ha^−1^)	(3)	[25]
$NHI=\frac{Seed\;N\;uptake}{Total\;N\;uptake}$	(4)	[25]
$NUtE=\frac{Seed\;Yield\;}{Total\;N\;uptake}$	(5)	[25]
$AGDD_{n}=\overset{n}{\underset{i=1}{\sum }}\left(\frac{T_{\max}+T_{\min}}{2}\right)-T_{\;\mathrm{base}}$ ^1^	(6)	[30]

^1^ Tmax is the highest daily temperature value. Tmin is the lowest daily temperature value. Tbase for flax is 5 °C [30].

## Data Availability

All data generated or analyzed during this study are included in this published article. Further inquiries can be addressed to the corresponding author.

## Abbreviations

N, Nitrogen; AGDDs, Accumulated Growth Degree Days; DAS, Days After Sowing; NHI, Nitrogen Harvest Index; NUtE, Nitrogen Utilization Efficiency; DM, Dry Matter; LAI, Leaf Area Index; ANU, Above-ground Nitrogen Uptake; SNU, Seed Nitrogen Uptake; TNU, Total Nitrogen Uptake.
